# Fatal *Toxoplasma gondii* Dissemination in a Heart Transplant Recipient: Description of a Case

**DOI:** 10.1155/2012/524279

**Published:** 2012-08-05

**Authors:** S. Mastrobuoni, A. M. Dell'Aquila, J. Herreros

**Affiliations:** ^1^Department of Cardiovascular Surgery, University Hospital of Navarre, Avenida Pio XII, 31008 Pamplona, Spain; ^2^Department of Cardiac Surgery, University of Ottawa Heart Institute, 40 Ruskin Street, Ottawa, ON, Canada K1Y 4W7

## Abstract

A 45-year-old heart transplant recipient presented with fever, anorexia, asthenia, and lethargy. She had received heart transplantation only 5 weeks earlier for primary systemic amyloidosis with severe cardiac involvement. Serum sodium was low, and tacrolimus through level was high. Blood cultures and serology tests for infection were negative, and atypical pneumonia was suspected. Despite broad antibiotic, antiviral, and antifungal treatment, the patient clinical condition rapidly deteriorated and she died within three days of admission. Postmortem examination revealed a disseminated *Toxoplasma gondii* infection as a result of donor (+)/recipient(−) mismatch for *Toxoplasma* serology. Although very rare, toxoplasmosis in heart transplant recipient should be suspect in case of neurological deficit and respiratory symptoms. Prophylaxis treatment is recommended in case of mismatch.

## 1. Case Description

A 45-year-old woman presented to our heart transplant outpatient clinic complaining of anorexia, asthenia, and lethargy. She denied shortness of breath, fever, chills, cough, or any other symptom.

She had undergone orthotopic heart transplantation 5 weeks earlier for end-stage heart failure due to primary systemic amyloidosis (amyloid light-chain, AL) with severe cardiac involvement with restriction diagnosed only few weeks before. Her past medical history was only significant for pulmonary tuberculosis treated 20 years before. Before the transplant, she was assisted with an Extracorporeal Membrane Oxygenator (ECMO) device during 35 hours due to unstable clinical conditions, and she was then transplanted on an urgent basis. The donor was a 50-year-old woman died of cerebral haemorrhage. There was a mismatch between donor and recipient for Epstein-Barr virus and *Toxoplasma* but not for *Cytomegalovirus*. The postoperative course after the transplant was uneventful, and the patient was discharged home on day 15 postoperatively. The first two endomyocardial biopsies were normal.

On physical examination, the patient had a temperature of 38.5°C, heart rate of 110, blood pressure of 120/80 mmHg, respiratory rate of 20, grossly normal neurological status without any deficit, a normal cardiac auscultation, some bilateral pulmonary basal crackles, an unremarkable abdomen, and a mild peripheral oedema.

She was on tacrolimus 2 mg/day, mycophenolate mofetil 2000 mg/day, prednisone 7,5 mg/day, and aspirin 100 mg/day. She was not receiving any antibiotic prophylaxis. She had had tolerance induction therapy with two doses of daclizumab (1 mg for kg of body weight) on day 1 and 14 postoperatively. The patient was waiting for bone marrow autotransplantation as definitive treatment of her primary systemic amyloidosis.

Blood chemistry revealed a haemoglobin of 10 g/dL (normal: 12–14), WBC: 17000/uL and 83% neutrophils (normal WBC: 6000–9000, neutrophils 40–60%), sodium: 126 mEq/L (normal 135–145 mEq/L), normal renal function, a slightly increase in hepatic enzymes (GOT and GPT 76 and 75 resp.). Tacrolimus and MMF through levels were 31 ng/mL and 1,6 ug/mL, respectively. C-reactive protein was 6.3 mg/dL. Chest X-ray revealed a small left pulmonary basal consolidation, which was suspected of pneumonia. CT-scan of the thorax showed scars and bronchiectasis in the left pulmonary base but did not confirm pneumonia.

On admission, suspension of tacrolimus, correction of hyponatremia with saline solution, and antimicrobial treatment with levofloxacin 500 mg iv/day, ceftriaxon 2 g iv/day, and amphotericin B 250 mg iv/day were started. Blood and sputum cultures were negative for bacteria and viruses. Serology for *Toxoplasma* as well as *Legionella pneumophila* persisted negative.

After an initial mild improvement of her symptoms, on the third day of admission the patient complained of shortness of breath at rest and a decreased level of conscience. Physical examination revealed increased Respiratory Rate, blood pressure of 100/60 mmHg, heart rate of 120, reduced air entry on both lungs, and peripheral hypoperfusion. Arterial blood gas revealed a pO_2_ of 57 mmHg, pCO_2_ of 24 mmHg, Sodium of 125 mEq/L, and a lactate concentration of 2.6 mmol/L. Vancomycin, trimethoprim/sulfamethoxazole, and acyclovir intravenously were added to the treatment under the suspicion of atypical pneumonia. Repeated serology for *Toxoplasma* and *Legionella* still resulted negative.

**Figure 1:**
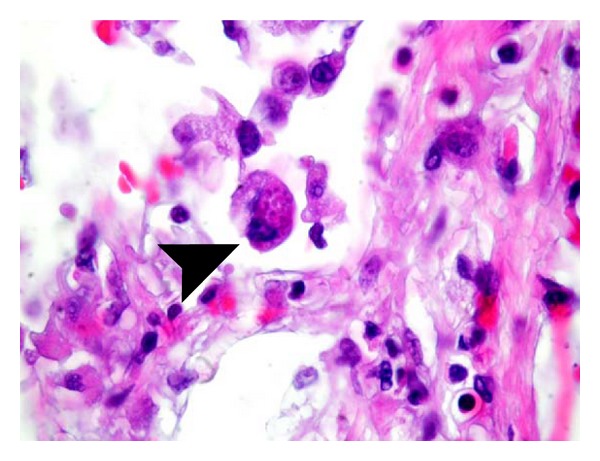
Cardiac section stained with hematoxylin/eosin showing interstitial and intra-alveolar infiltrates of lymphocytes and monocytes/macrophages containing *Toxoplasma* trophozoites.

The patient's clinical condition rapidly worsened with respiratory failure that required mechanical ventilation, liver and renal failure that required replacement therapy. The patient finally died of multiorgan failure the same day. Postmortem examination revealed a disseminated toxoplasmosis with involvement of the heart, lungs, and liver. Microscopic analysis of pulmonary and cardiac sections (Figure 1) stained with hematoxylin/eosin revealed interstitial and intra-alveolar infiltrates of lymphocytes and monocytes/macrophages containing *Toxoplasma* trophozoites. Culture of tissue sample from lungs was negative for bacterial and CMV infection.

## 2. Discussion

We have presented a fatal case of disseminated *Toxoplasma *infection in a heart transplant recipient. Although the seroprevalence of *Toxoplasma* is high in the adult population, around 75% in Europe [1], this recipient was negative for both IgM and IgG. Transmission of *Toxoplasma* can occur in the setting of transplantation of organs from a seropositive donor into a seronegative recipient. Heart transplantation is at risk for parasitic transmission as tissue cysts are found in the myocardium. Transmission of *Toxoplasma* in seronegative recipients exceeds 80% [1] but the clinical presentation, the toxoplasmosis, is very rare. In a series of 620 heart transplants at Stanford University, among 16 seronegative recipients only 4 cases of infection were observed [2]. Furthermore, due to a very low incidence of *Toxoplasma* infection in heart transplant recipients in Spain in the last 10 years (0.6%) [3] and none incidence of opportunistic infections such as *Toxoplasma* or *Pnuemocystis* in our Centre, the patient was not receiving antimicrobial therapy. Indeed, this is the first case in our experience. Herein, this patient acquired the infection with the donor heart but increased immunosuppression, as evidenced by the high tacrolimus through levels, possibly facilitated the dissemination of the *Toxoplasma* [4].

The most common presentation of toxoplasmosis is the encephalitis and typically occurs in the first three months after transplantation. Our patient indeed presented 5 weeks postoperatively with lethargy that was initially attributed to the hyponatremia and toxic levels of Tacrolimus. Toxoplasmosis can also present as pneumonia, acute respiratory failure, and hemodynamic compromise [5]. *Toxoplasma* pneumonia should be suspected when bacterial infection is ruled out.

Diagnosis of toxoplasmosis can be made with serological and molecular methods, but confirmation of the parasite in immunocompromised patients may require biopsy samples of infected organs, especially heart and lung, with demonstration of trophozoites, as the tissue cysts persist in the organ for many years after acute infection and thus are not diagnostic [1]. Moreover, the presence of tissue cysts in routine endomyocardial biopsies is an occasional finding. This patient never presented seroconversion, and the two previous biopsies were also negative for *Toxoplasma*.

Observational studies suggest that trimethoprim/sulfamethoxazole may be effective even in cardiac recipients in preventing toxoplasmosis, and prophylaxis for seronegative recipients is recommended [1]. Treatment of disseminated infection includes reduction of the immunosuppressive therapy and a combination of antimicrobial agents including pyrimethamine and sulfonamide or clindamycin. Although correct treatment, toxoplasmosis can still cause a devastating disease such as in our patient [6].

In conclusion, in transplanted patients with neurological and respiratory symptoms, *Toxoplasma* infection should be suspected especially in presence of donor/recipient mismatch. Prompt treatment, even without confirmation of *Toxoplasma*, should be started, as the toxoplasmosis can be rapidly fatal.
